# *Aspergillus oryzae* Fermented *Plumula Nelumbinis* Against Atopic Dermatitis Through AKT/mTOR and Jun Pathways

**DOI:** 10.3390/ph18010020

**Published:** 2024-12-27

**Authors:** Fengfeng Chen, Jing Liu, Xinwei Yu, Honglei Jia, Cheng Yang, Bingtian Zhao

**Affiliations:** 1 Key Laboratory of Synthetic and Biological Colloids, Ministry of Education, School of Chemical and Material Engineering, Jiangnan University, Wuxi 214122, China; cff@jiangnan.edu.cn (F.C.); lj15735649029@163.com (J.L.); 17361731165@163.com (X.Y.); cyang@jiangnan.edu.cn (C.Y.); 2 Shanghai Fulai BioHighTech Co., Ltd., Shanghai 201400, China; gracexiaobai@126.com

**Keywords:** *Plumula Nelumbinis*, alkaloid, fermentation, atopic dermatitis, network pharmacology, mechanism

## Abstract

**Background/Objectives:** Atopic dermatitis (AD) is a chronic inflammatory skin disorder that has attracted global attention, and alkaloids from *Plumula Nelumbinis* have been shown to have anti-inflammatory activity. Fermentation has been used for the structural modification of natural compounds to improve bioavailability and activity, but the AD therapeutic efficacy and mechanism of the fermented *Plumula Nelumbinis* (FPN) are still unclear. **Methods:** The potential targets of FPN for AD were preliminarily screened using network pharmacology, and then PCR and WB were used to prove the therapeutic effect of FPN in AD. **Results:** Network pharmacology indicated that mTOR and Jun were key targets for AD. The experiments in vitro showed that FPN could effectively block AKT/mTOR and AKT/Jun-mediated inflammatory signaling pathways. Moreover, FPN can also alleviate SDS-induced inflammation in zebrafish. It is also found that the anti-inflammatory activity of *Plumula Nelumbinis* was enhanced by *Aspergillus oryzae* fermentation, and the oil phase of the fermentation product showed better activity, which may be due to microbial fermentation changing the structure of the original alkaloids. **Conclusions:** This study elucidated the potential mechanisms of alkaloids derived from fermented *Plumula Nelumbinis* against AD; it may also provide a scientific basis for the development of new drugs for AD.

## 1. Introduction

Atopic dermatitis (AD) is a long-lasting inflammatory skin condition marked by specific skin rashes and severe itching, which significantly impacts the patient’s life [1]. Genetic, physiological, and environmental factors are considered key contributors to the development of AD, and improving the physiological immune imbalance is becoming an important approach for treating this condition [2,3,4]. Epidemiological findings showed that the prevalence of AD is 10–20% among children and 1–5% among adults in developed countries, and the prevalence in males is 30% higher than that in females [5,6]. Additionally, the associated healthcare costs for AD patients reached USD 157.7 per month [7]. Therefore, there is an urgent need to strengthen the effective disease management of AD to improve the lives of patients and reduce the economic burden of society. Currently, conventional drugs used in clinical treatment of AD include corticosteroids, antihistamines, and immunosuppressants. However, long-term use of these drugs poses serious issues such as severe side effects and a high relapse rate [8]. Hence, there is an urgent need to find new, safe, and effective therapeutic options. In recent years, natural products have garnered widespread attention in the field of atopic dermatitis treatment due to their wide availability, high safety profile, and potential multi-target synergistic effects [9]. Meanwhile, polysaccharides [10], flavonoids [11], alkaloids [12], and fatty acids [13] all showed positive effects in the treatment of AD.

The *Plumula Nelumbinis* is the dried young leaves and radicle from the mature seeds of *Nelumbo nucifera* Gaertn., traditionally used in Chinese medicine for centuries [14]. It contained various compounds, among which alkaloids are particularly notable. The alkaloids in *Plumula Nelumbinis* mainly include Liensinine, Isoliensinine, and Neferine [15,16]. These alkaloids possess anti-inflammatory [17] and antioxidant effects [18], which are vital for treating conditions like atopic dermatitis. Furthermore, Neferine from *Plumula Nelumbinis* has been preliminarily shown to alleviate AD by inhibiting the expression of inflammatory factors and improving skin lesions in mouse models [19]. These findings provide further evidence that the alkaloids in *Plumula Nelumbinis* could be promising candidates for treating AD. Furthermore, these alkaloids need to undergo preliminary metabolism in the body before they can be effectively utilized. Lin et al. indicated that alkaloids from *Plumula Nelumbinis* are primarily bioavailable through demethylation in Caco-2 cells [20]. Han et al. found that in mice, alkaloids undergo processes, such as hydroxylation, demethylation, glucuronidation, and sulfation, before they can be effectively utilized [21]. Hence, it is essential to modify the chemical structure of alkaloids, altering their physicochemical properties to improve their solubility and bioavailability.

Microbial fermentation techniques have been widely used in the processing of traditional Chinese medicinal herbs to enhance or generate new pharmacological effects. During the fermentation process, various enzymes are produced, which can interact with the alkaloids in *Plumula Nelumbinis* to achieve structural modifications. Furthermore, the fermentation process may lead to the production of new active metabolites, which may further enhance their anti-inflammatory, immunomodulatory, and other therapeutic activities for treating atopic dermatitis (AD). *Morinda citrifolia* [22] and *Portulaca oleracea* L. [23] fermented by *Lactobacillus plantarum* can regulate the immune balance and skin barrier of AD. Meanwhile, the rice bran [24] fermented by *Lactobacillus rhamnosus* and *Pichia deserticola* can reduce the expression of inflammatory factors in AD mice. These fermented products have shown positive effects in the treatment of AD. Therefore, microbial fermentation exhibits great promise for developing more effective drugs for the management of AD.

In our previous research, it was found that *Aspergillus oryzae* can degrade the Isoliensinine and Neferine of *Plumula Nelumbinis* into new alkaloid derivatives. The fermentation products (FPN) significantly inhibited atopic dermatitis induced by TNF-α/IFN-γ in HaCaT [25]. However, the mechanism of its active ingredients in treating AD remains unclear. Network pharmacology, as an emerging research method, can comprehensively elucidate the molecular mechanisms of drugs by constructing compound-target-disease networks [26]. This holistic approach is particularly applicable to complex multi-genetic, multi-target diseases like AD. Molecular docking technology plays a key role in network pharmacology studies [27]. Simulating the interactions between small molecule compounds and large molecular receptors helps identify the active ingredients that strongly interact with AD-related targets, providing valuable guidance for subsequent experimental studies and drug development.

In this study, network pharmacology was applied to screening the core targets of alkaloid derivatives in the fermentation products for AD treatment. The binding affinity between six alkaloids and the core targets was calculated by molecular docking. Based on these findings, the dermatitis model was established in HaCaT using TNF-α and IFN-γ (TI), and the predicted targets were determined through WB and PCR. Meanwhile, zebrafish were employed to assess the anti-inflammatory and alleviating effects of FPN.

## 2. Results

### 2.1. Potential Targets of FPN for AD Treatment

**Figure 1:**
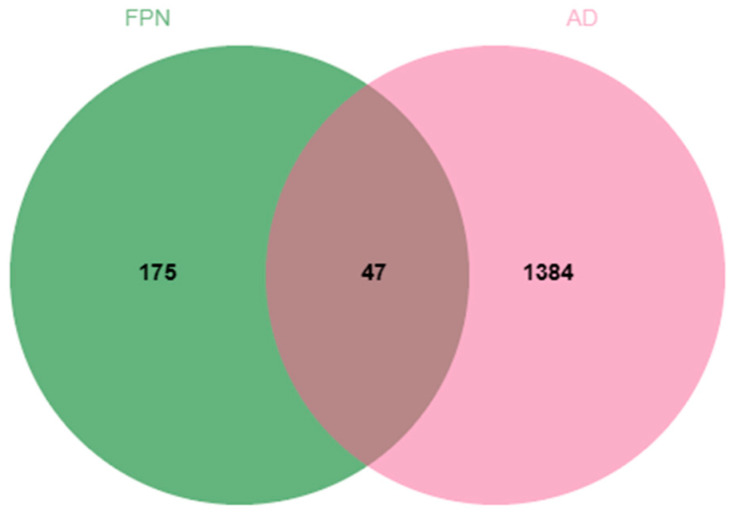
The Venn diagram of potential targets for FPN in the treatment of atopic dermatitis.

From the compound target database, a total of 222 target genes for the six compounds were obtained. Using “dermatitis” as the keyword, we retrieved 1431 targets from the disease databases. Meanwhile, the 47 common targets of FPN and AD were acquired by Venn diagram online at Venny 2.1 (Figure 1).

### 2.2. “Compounds–Target–AD” Network

**Figure 2:**
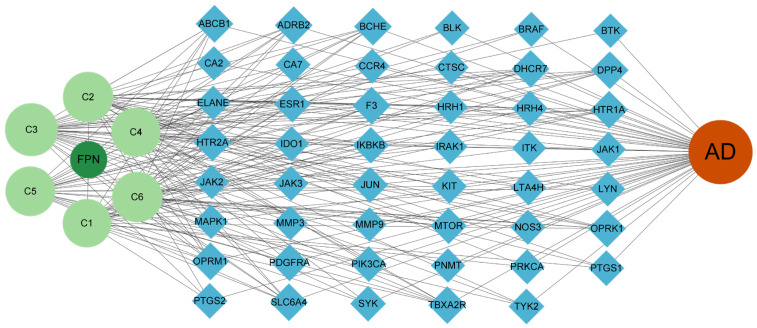
Compounds–target–AD network of the potential therapeutic targets and active components of FPN (C1-C6). C1~C6 represent Armepavine, N-Demethylcolletine, N-Methylcoclaurine, Neferine, Liensinine, and Isoliensinine, respectively.

The network diagram of “Compounds–Target–AD” was established (Figure 2). The six green circles represent the major alkaloidal components, and the blue lozenges represent potential targets for their action. The interactions between compounds and targets were represented by lines, with a greater number of lines originating from a compound, which indicated a potential interaction with more targets. The result showed that N-Methylcoclaurine, N-Demethylcolletine, and Isoliensinine were the top three compounds with more targets. Notably, N-Methylcoclaurine and N-Demethylcolletine were products obtained by fermentation, suggesting that the fermented products may have superior anti-inflammatory effects.

### 2.3. PPI Network Diagram

**Figure 3:**
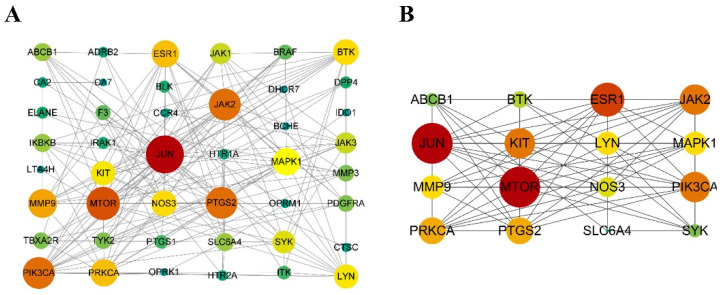
PPI network of the following: (**A**) Common targets; (**B**) Core targets.

The 47 shared targets were imported into the String database, and the proteins were then imported into Cytoscape 3.9.1 to generate a PPI diagram. The network diagram contained 44 nodes and 171 edges, where nodes represent target proteins and edges reflect interactions between proteins. As illustrated in Figure 3A, the top five nodes ranked by degree value were Jun, mTOR, PIK3CA, PTGS2, and JAK2, and these proteins are mainly associated with inflammation, cell survival, and apoptosis. The target proteins were filtered based on the criteria of Degree > 7 (median), Betweenness Centrality > 0.01688 (median), and Closeness Centrality > 0.43017 (median), resulting in 32 core target proteins. These core proteins were then imported into the String to generate a PPI network graph (Figure 3B). This network graph contained 16 nodes and 75 edges, and the top three core targets were mTOR, Jun, and ESR1.

### 2.4. GO and KEGG Enrichment Analysis

**Figure 4:**
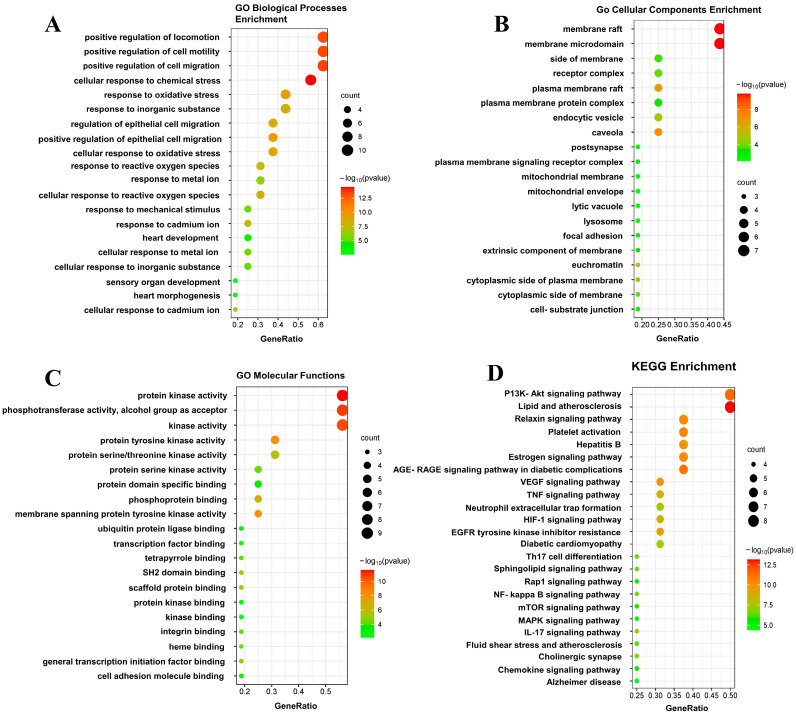
GO and KEGG enrichment analyses of potential core targets: (**A**) The top 20 enrichment terms for BP. (**B**) The top 20 enrichment terms for CC. (**C**) The top 20 enrichment terms for MF. (**D**) The top 20 KEGG pathways.

The terms 341 BP, 22 CC, and 21 MF were enriched in the GO analysis, and the top 20 terms were shown in Figure 4. The BP terms primarily involved cellular responses to chemical stress, responses to oxidative stress, and positive regulation of locomotion (Figure 4A). The CC terms were mainly related to membrane rafts, membrane microdomain, and receptor complex (Figure 4B). The MF terms were associated with protein kinase activity, phosphoprotein binding, and kinase activity (Figure 4C). According to the KEGG analysis, the genes were mainly implicated in pathways related to inflammation, such as the PI3K-AKT, TNF, and NF-kappa B (Figure 4D).

### 2.5. Molecular Docking Results

mTOR and Jun were identified as the top two core targets in the PPI network, so the interaction between the six compounds and these two targets was further analyzed by molecular docking. The proteins were used as protein receptors, while six compounds were utilized as molecular ligands for docking. The binding energies of the six compounds with the core targets were less than −5 kcal/mol (Table 1), suggesting that the compounds spontaneously and stably bind to the receptor proteins at the molecular level [28]. Among these compounds, Neferine and Liensinine exhibited lower binding energy to both proteins.

**Figure 5:**
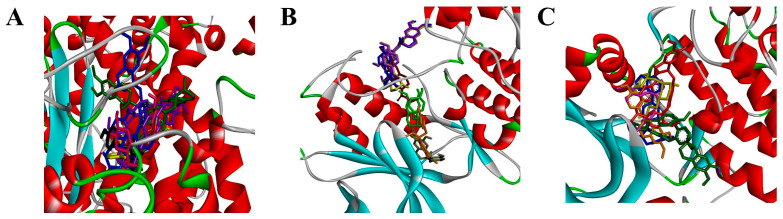
Molecular docking conformations of six compounds with the following: (**A**) mTOR; (**B**) AKT; (**C**) Jun.

The interaction patterns between the compound and the protein are depicted in Figure 5, and the results showed that different compounds had different binding conformations to the same protein. The formation of non-covalent bonds, such as hydrogen bonds, van der Waals forces, and electrostatic interactions, is the key factor influencing the binding of compounds and proteins. In the mTOR docking assay, Neferine, Liensinine, and Isoliensinine exhibited better binding affinity than the control. Furthermore, all the compounds except N-Demethylcolletine have the common amino acid residue of GLY2142. As for compound N-Demethylcolletine, compared with control, it contains the common amino acid residues of LEU1936, MET2199, GLN2200, and GLY2203 (Table S1 and Figure S1). In the Jun docking assay, Neferine, Liensinine, and Isoliensinine exhibited better binding affinities (<−10 kcal/mol) than the control and are similar to the mTOR docking result. These three compounds have the common amino acid residues of THR103, LYS106, and TYR240, and they have the same amino acid residue of ARG230 as that of the control group. The other three compounds contain the common amino acid residues of GLY73, and SER193 (Table S1 and Figure S2). The AKT docking results indicated that, compared with the other three compounds, Neferine, Liensinine, and Isoliensinine also had a lower energy score. All the compounds except N-Methylcoclaurine have the common amino acid residue of GLY159 (Table S1 and Figure S3). Anyway, all six compounds possess relatively low bingding energy and likewise have the potential to inhibit the those mTOR, Jun, and AKT proteins.

### 2.6. Effect of FPN on Gene Expression

**Figure 6:**
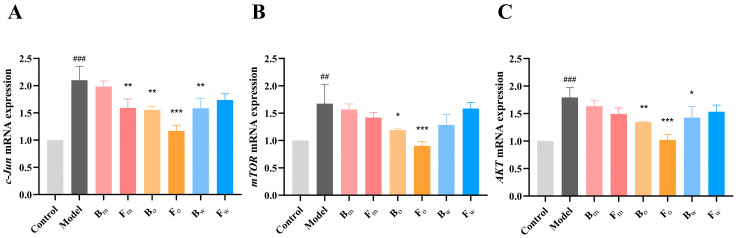
The gene expression of the following: (**A**) c-Jun; (**B**) mTOR; (**C**) AKT. ## *p* < 0.01, ### *p* < 0.001 vs. control group, * *p* < 0.05, ** *p* < 0.01, *** *p* < 0.001 vs. model group.

In this study, the potential core protein targets related to dermatitis treatment were gained through network pharmacology. To further investigate the regulatory effects of FPN on these targets, PCR technology was employed to assess the gene expression of these targets, providing important insights into the action mechanism of FPN. The gene levels of AKT, mTOR, and c-Jun were significantly increased in the model group, which demonstrated that the atopic dermatitis microenvironment had been successfully created. The expression of c-Jun in the FPN group (Fm, Bo, Fo, and Bw) was significantly downregulated, but the pre-fermentation extract (Bm) and the post-fermentation aqueous phase (Fw) did not show inhibitory effects (Figure 6A). Moreover, only the post-fermentation aqueous and organic phases were able to significantly reduce the gene expression levels of mTOR and AKT (Figure 6B,C), suggesting that fermentation enhanced the anti-inflammatory properties of *Plumula Nelumbinis*.

### 2.7. Effect of FPN on Protein Expression

**Figure 7:**
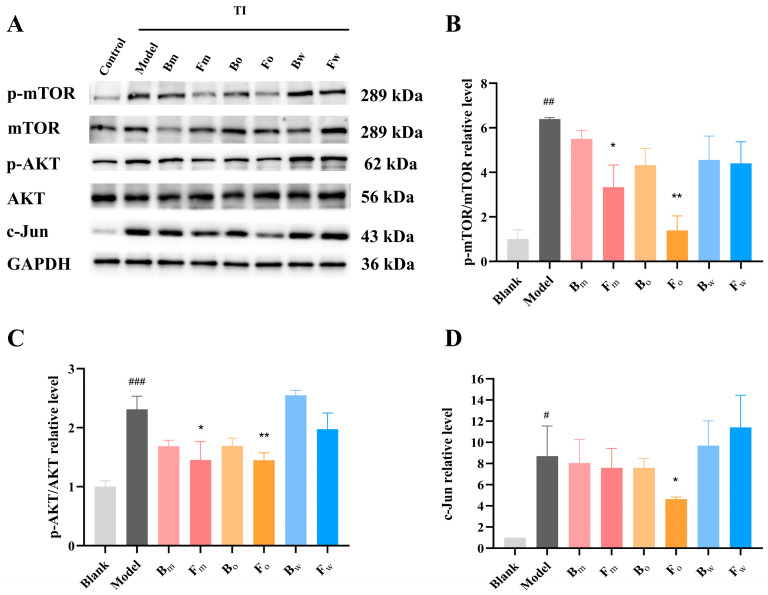
Effects of FPN on protein expression: (**A**) Western blot bands; (**B**) Quantitative analysis of the relative expression of p-mTOR/mTOR; (**C**) p-AKT/AKT/; (**D**) c-Jun. # *p* < 0.05, ## *p* < 0.01, ### *p* < 0.001 vs. control group, * *p* < 0.05, ** *p* < 0.01 vs. model group.

To validate the effect of FPN on the core targets (mTOR and c-Jun) in the PPI network, as well as the PI3K/AKT signaling pathway identified by KEGG enrichment analysis, the protein expression was examined using WB. TI stimulation results in elevated phosphorylation of AKT and mTOR proteins, and this phosphorylation modification alters the activity of the proteins, which in turn promotes the development and progression of inflammation. The findings indicated that FPN had a certain regulatory effect on the proteins mentioned above (Figure 7), and only the post-fermentation (Fm) and its oil phase (Fo) showed a significant downregulation effect.

### 2.8. The Effect of FPN on Neutrophils in Zebrafish

**Figure 8:**
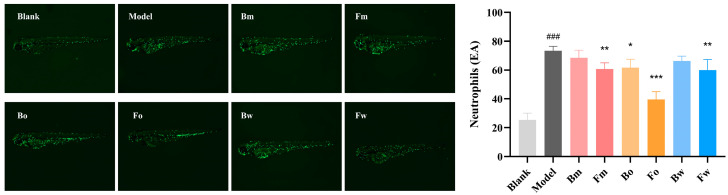
Effect of FPN on neutrophil recruitment. ### *p* < 0.001 vs. control group, * *p* < 0.05, ** *p* < 0.01, *** *p* < 0.01 vs. model group.

Zebrafish share over 70% of their genetic material with humans, which makes them an excellent model for studying human diseases. Their genetic similarity, transparency of embryos, rapid reproduction, and ease of manipulation provide valuable insights into disease mechanisms, drug testing, and developmental biology [29]. To investigate the potential therapeutic effects of different fractions on AD, an inflammation model induced by SDS in zebrafish was established. SDS is a well-known agent for inducing skin inflammation and mimicking features of AD in zebrafish, making it an appropriate model for evaluating anti-inflammatory activity. Upon exposure to SDS, zebrafish larvae exhibited increased neutrophil infiltration, which is a characteristic of inflammatory responses observed in AD (Figure 8). Treatment with FPN significantly reduced the number of neutrophils (except for Bm and Bw), with Fo exhibiting the strongest anti-inflammatory effect, followed by Fw and Fm.

## 3. Discussion

The inflammatory response is a vital factor in the pathophysiology of AD. A large number of T lymphocytes and eosinophils tend to accumulate and activate in AD patients, which affects the occurrence and progression of AD by secreting cytokines and chemokines [30,31]. Consequently, the current treatment strategies for AD are mainly focused on anti-inflammatory interventions, which aim to suppress hyperactive immune responses and repair the skin barrier. *Plumula Nelumbinis* is a traditional herbal medicine commonly used for calming the mind and stopping bleeding. A total of 130 natural compounds have been isolated from it, among which alkaloids are important pharmacological components [32]. Although pharmacological research has thoroughly explored the anti-tumor, anti-depressant, and hypotensive effects and mechanisms of alkaloids, the investigation of their anti-inflammatory properties is very limited. Notably, recent studies have highlighted the anti-inflammatory effects of alkaloids such as higenamine and nuciferine, which have been shown to alleviate symptoms of arthritis and LPS-induced pulmonary injury, respectively [33,34]. These findings suggested that the alkaloids of *Plumula Nelumbinis* possessed considerable anti-inflammatory activity, making it a promising candidate for AD treatment. Moreover, our previous research showed that *Plumula Nelumbinis* and its fermentation products possessed marked anti-inflammatory and antioxidant properties, and the fermentation products displayed better efficacy. The objective of this study is to explore the mechanisms of *Plumula Nelumbinis* in treating AD.

Network pharmacology, as an innovative research approach, can reveal the complex mechanism of drugs by systematically integrating multiple relationships between drugs, targets, and diseases. Network pharmacology emphasizes the multi-target and multi-pathway nature of drugs, offering a more comprehensive and systematic view of their effects [35]. In our study, network pharmacology revealed 47 potential targets of FPN for AD, and Jun, mTOR, and PIK3CA were the top three targets in PPI. Jun is a key member of activator protein-1 (AP-1), which is critical in modulating cellular activities like proliferation, apoptosis, and inflammation. In various inflammatory diseases, Jun can upregulate the level of pro-inflammatory cytokines and chemokines, thereby driving the initiation and persistence of inflammation [36]. Additionally, the AP-1 complex is highly expressed in lesional skin of atopic dermatitis (AD), and T-5224 (an AP-1 inhibitor) has been shown to alleviate AD symptoms in mice by inhibiting Jun expression [37]. mTOR is a serine/threonine kinase that senses extracellular nutrients, growth factors, and energy status. mTOR pathway dysregulation can cause various conditions, including psoriasis [38], acne [39], and atopic dermatitis [40]. PIK3CA, a member of the PI3K family, is found in various skin cells and contributes to skin-related pathological processes. Moreover, PI3K can regulate the downstream mTOR pathway by activating phosphorylated AKT proteins [41].

KEGG enrichment analysis has demonstrated significant enrichment of several pathways associated with inflammatory responses. The activation of pathways such as PI3K-AKT, TNF, NF-κB, and mTOR was shown to be closely related to alterations in immune cell function. These findings indicated that FPN may exert its therapeutic effect on AD by modulating multiple inflammatory signaling pathways. PI3K/AKT signaling is a potential pathway targeted by FPN in AD treatment. The PI3K/AKT pathway belongs to the serine/threonine protein kinase family and regulates several downstream proteins, including the PI3K/AKT/mTOR and the PI3K/AKT/c-Jun pathway [42]. Aberrant activation of Th2 is an important trigger for the pathogenesis of AD, and mTOR has been shown to regulate Th2 activation by AKT, which makes the PI3K/AKT/mTOR pathway an effective strategy for AD [43]. The progression of AD can be exacerbated by the NF-κB activation. It has been demonstrated that NF-κB suppression by resveratrol results in the alleviation of skin lesions in dermatitis mice, with fewer T cells in the skin [44]. In addition, previous studies have shown that Jun and AKT are also the core targets of Cortex Dictamni, *Scutellaria baicalensis*, and *Raphanus sativus* in the treatment of AD, and their importance for AD treatment has been demonstrated by mice experiments [45,46].

To further validate the network pharmacology results, PCR and WB were used to detect the expression of core targets (mTOR, AKT, c-Jun), respectively. TNF-α and IFN-γ (TI) are commonly used immune factors in dermatitis models, which can mimic the occurrence and progression of dermatitis by activating the immune response and disrupting the skin barrier function, thereby providing a reliable experimental model for related research [47]. In the microenvironment of dermatitis, the expressions of mTOR, AKT, and c-Jun in HaCaT were increased to about 1.6, 1.73, and 2.0 times, respectively. Bo and Fo exhibited a reduction in mTOR and AKT mRNA expression. For c-Jun, Fm, Bo, Fo, and Bw were all demonstrated to inhibit expression. Moreover, the post-fermentation oil phase (Fo) had the strongest inhibitory effect on the expression of these proteins. WB analysis further showed that Fo inhibited the activation of mTOR, AKT, and c-Jun pathways. In a previous study, D-mannose was found to be able to inhibit the activation of the mTOR pathway, thereby improving the inflammatory response in AD mice, which is similar to our findings [48]. Moreover, another study evaluated the AD therapeutic potential of *Portulaca oleracea* L. (PO) and its fermentation products (FPO) and found that the antioxidant and anti-inflammatory abilities of FPO were significantly improved, which is consistent with the results of our study [23]; this suggests that fermentation could enhance the biological activity of Chinese herbal medicines. Then, we investigated the anti-inflammatory and soothing effects of FPN in vivo by assessing the number of neutrophils in zebrafish. The results revealed that both the pre-fermentation Bo and post-fermentation Fm, Fo, and Fw could significantly inhibit neutrophil recruitment, and Fo also exhibited the best anti-inflammatory effect. While these studies have initially uncovered the mechanism of FPN in treating AD, there are limitations in our research. While this study primarily examines the alkaloid components of *Plumula Nelumbinis* before and after fermentation to explore their therapeutic mechanism for AD, other compounds may also contribute to the treatment of AD. Additionally, further pharmacological experiments in vivo and in vitro are essential to clarify the underlying mechanisms.

## 4. Materials and Methods

### 4.1. Materials

The keratinocytes (HaCaT) were purchased from the BNCC (BNCC339817, Beijing, China). DMEM, penicillin–streptomycin solution was obtained from Gibco (Grand Island, NY, USA). AKT, p-AKT, mTOR, p-mTOR, and GAPDH antibodies were bought from CST (Danvers, MA, USA). The Secondary antibodies were purchased from Beyotime Biotechnology (Shanghai, China). The fermented *Plumula Nelumbinis* was prepared according to the previous study [25], and Bm (before fermentation), Fm (after fermentation), Bo (oil phase before fermentation), Fo (oil phase after fermentation), Bw (aqueous phase before fermentation), and Fw (aqueous phase after fermentation) were prepared separately for subsequent studies.

### 4.2. Prediction of Drug and Disease Targets

A systematic search was conducted in the TCMSP and Swiss Target Prediction to identify the protein targets of the six compounds. Using “dermatitis” as the keyword, we searched for protein targets associated with AD in the GeneCards, OMIM, TTD, and DrugBank databases [49,50]. Venny 2.1.0 was used to analyze shared targets between compounds and dermatitis.

### 4.3. Network Construction

The common protein interactions were analyzed using the STRING database. The species condition was limited to “Homo sapiens” and the highest confidence baseline was set to 0.700, excluding free nodes. Then, the results were imported into Cytoscape to realize the visualization. The related parameters of each target protein on the PPI network were analyzed by using the tool “Analyze Network” in the Cytoscape 3.9.1 software [51], and the “compounds–target–AD” network was constructed.

### 4.4. Gene Ontology (GO) and Kyoto Encyclopedia of Genes and Genomes (KEGG) Enrichment Analysis of Core Targets

Gene Ontology (GO) and Kyoto Encyclopedia of Genes and Genomes (KEGG) enrichment analysis was conducted by the DAVID database [52] with the species set to “*Homo sapiens*”. The GO categories cellular component (CC), biological process (BP), and molecular functioning (MF) were selected for enrichment analysis. Then, the top ten GO terms and the top twenty KEGG pathways were visualized.

### 4.5. Molecular Docking

The 3D structures of mTOR (PDB ID: 4JSV), Jun (PDB ID: 2P33), and AKT (PDB ID: 3OCB) were retrieved from the Protein Data Bank (PDB) and PubChem, respectively. Subsequently, we import these proteins with 3D structure into AutoDock Vina, perform desolvation and hydrogenation, and then designate the target proteins as receptors. Similarly, the active compounds were pre-processed with full hydrogenation and designated as ligands. The compounds for each target with a grid box size of 40 × 40 × 40. The binding affinity between receptors and the targets was evaluated by molecular docking using Autodock Vina 2.0, and it was visualized with Discovery studio client 2019 [53].

### 4.6. RT-qPCR

HaCaTs were cultured in DMEM with 10% FBS and 1% penicillin–streptomycin at 37 °C in 5% CO_2_ and seeded into 6-well plates at 5 × 10^5^ cells/mL. When the cells reached 80% confluence at the bottom of the culture dish, the samples were added to the corresponding wells. The cells were divided into blank (DMEM), model (DMEM containing TI), Bm (DMEM containing TI and Bm), Fm (DMEM containing TI and Fm), Bo (DMEM containing TI and Bo), Fo (DMEM containing TI and Fo), Bw (DMEM containing TI and Bw), and Fw (DMEM containing TI and Fw). After 24 h, the total RNA was extracted using a Trizol reagent. RT-qPCR measurements were carried out utilizing the SYBR Green Master Mix. The 2^−ΔΔCt^ method was used to quantify mRNA levels and normalized to GAPDH [54], with primer sequences listed in Table 2.

### 4.7. Western Blot Analysis

The cells were grouped and treated in the same way. After 24 h, the cells were collected by centrifugation, and the RIPA lysate was added to the cells to prepare the protein. Mix the protein solution with the loading buffer and denature the protein by heating. Proteins were separated using 10% SDS-PAGE gel and then transferred to PVDF membranes. After blocking with 5% BSA, The PVDF membrane is incubated first with the primary antibody, followed by the secondary antibody. Finally, the ECL reagent (Beyotime, Shanghai, China) was applied for 3 min to visualize the protein bands. Protein band intensity was analyzed using ImageJ 1.53 software.

### 4.8. The Anti-Inflammatory Activity Assay In Vivo

Sodium dodecyl sulfate (SDS) was used to establish an inflammatory stimulation model in zebrafish to preliminarily evaluate the anti-inflammatory effect of the drug. Wild-type AB strains of zebrafish 3 days after fertilization were selected, and they were randomly assigned to 6-well plates with 15 larvae/well. Then, they were treated with a culture medium containing FPN and SDS (sample group), a culture medium containing SDS (model group), and a culture medium (blank group), respectively. After 18 h, It was observed and photographed using a fluorescence microscope to accurately count the number of neutrophils in zebrafish.

### 4.9. Statistical Analysis

GraphPad Prism 8.0.2 was used for statistical analysis. Data are shown as mean ± standard deviation, and one-way ANOVA was applied to determine significant differences. *p*-value < 0.05 was considered statistically significant.

## 5. Conclusions

This study elucidated the impact and mechanism of FPN from *Plumula Nelumbinis* on AD, and the core targets were identified using network pharmacology and tested through experimental validation. AKT/mTOR and AKT/Jun signaling may be the crucial pathway for FPN to reduce the overactivated immune response and relieve AD. Notably, it was discovered that the fermented *Plumula Nelumbinis* showed better suppression of inflammatory pathways, especially in the Fo fraction. This work suggests that the alkaloid derivatives, which are produced by fermentation, have the potential for AD treatment and provide a scientific basis for the development of new drugs for AD.

## Figures and Tables

**Table 1:** The binding energy of compound and core targets.

Target	Affinity (kcal/mol)						
	Neferine	Liensinine	Isoliensinine	Armepavine	N-Demethylcolletine	N-Methylcoclaurine	Control
mTOR	−11.1	−11.2	−11.6	−8.9	−8.6	−7.4	−8.9
Jun	−10.4	−10.7	−10.1	−8.1	−9.1	−7.2	−7.6
AKT	−10.0	−9.9	−9.9	−7.9	−8.5	−7.6	−8.8

**Table 2:** Real-time polymerase chain reaction primers.

Gene	Primer	Sequence (5′-3′)	Length (bp)
Homo GAPDH	Forward	TCAAGAAGGTGGTGAAGCAGG	115
	Reverse	TCAAAGGTGGAGGAGTGGGT	
Homo AKT	Forward	ACACCAGGTATTTTGATGAGGAG	143
	Reverse	TCAGGCCGTGCCGCTGGCCGAGTAG	
Homo c-Jun	Forward	AACGTGACAGATGAGCAGGA	232
	Reverse	CTGGGTTGAAGTTGCTGAGG	
Homo mTOR	Forward	CCCCTTCACCAGTTTCCA	220
	Reverse	CAGCGAGTTCTTGCTATTCC	

## Data Availability

Data are contained within the article.

## Supplementary Materials

The following supporting information can be downloaded at: https://www.mdpi.com/article/10.3390/ph18010020/s1, Figure S1: The interaction of mTOR and six compounds; Figure S2: The interaction of Jun and six compounds; Figure S3: The interaction of AKT and six compounds; Table S1: Binding sites of six compounds for three targets.
