# Supplementary material for: Aspergillus oryzae Fermented Plumula Nelumbinis Against Atopic Dermatitis Through AKT/mTOR and Jun Pathways
Source: Pharmaceuticals (Basel). 2024 Dec 27;18(1):20. doi: 10.3390/ph18010020 (PMC11768159; doi:10.3390/ph18010020)
Supplement: Supplementary file 1 [file pharmaceuticals-18-00020-s001.zip › pharmaceuticals-3376237-supplementary.pdf]

# *Aspergillus oryzae* Fermented *Plumula Nelumbinis* Against Atopic Dermatitis Through AKT/mTOR and Jun Pathways

**Fengfeng Chen [1], Jing Liu [1], Xinwei Yu [1], Honglei Jia [2], Cheng Yang [1] and Bingtian Zhao [1,*]**

[1] Key Laboratory of Synthetic and Biological Colloids, Ministry of Education, School of Chemical and Material Engineering, Jiangnan University, Wuxi 214122, China; cff@jiangnan.edu.cn (F.C.); lj15735649029@163.com (J.L.); 17361731165@163.com (X.Y.); cyang@jiangnan.edu.cn (C.Y.)
[2] Shanghai Fulai BioHighTech Co., Ltd., Shanghai 201400, China; gracexiaobai@126.com
* Correspondence: btzhao@jiangnan.edu.cn

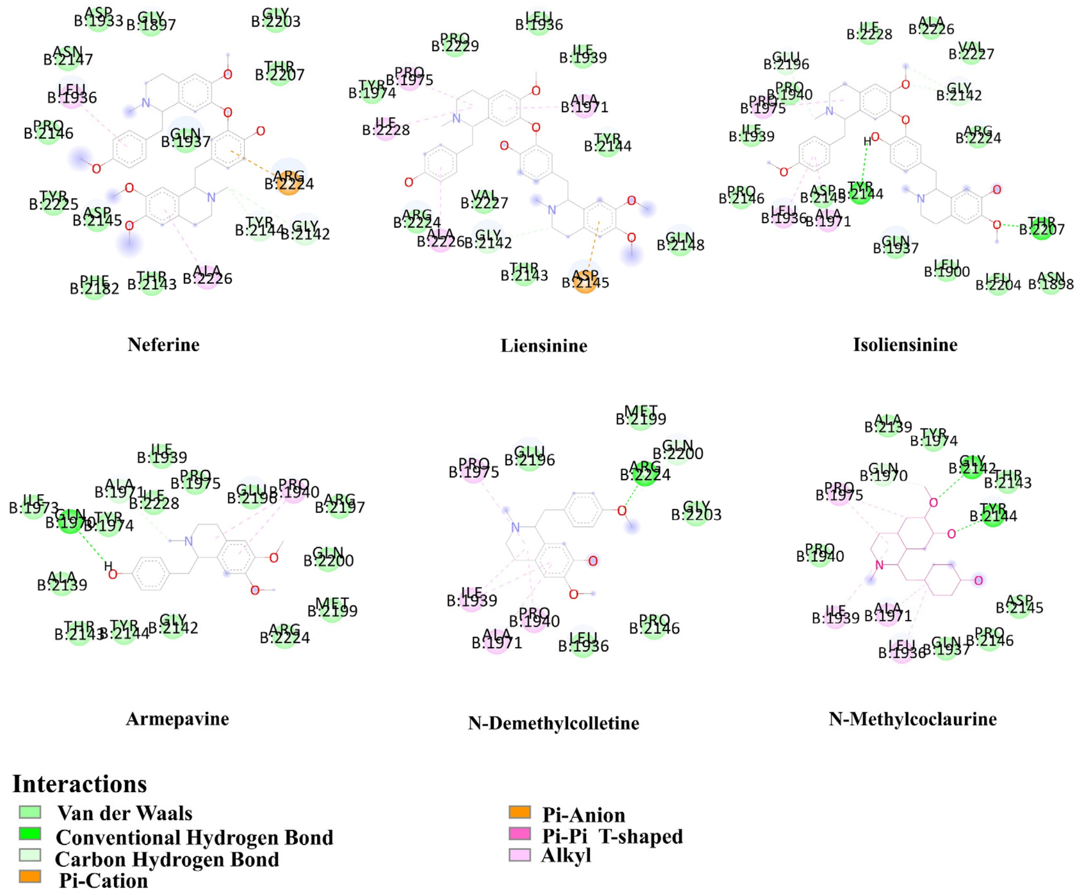


**Figure S1.** The interaction of mTOR and six compounds.

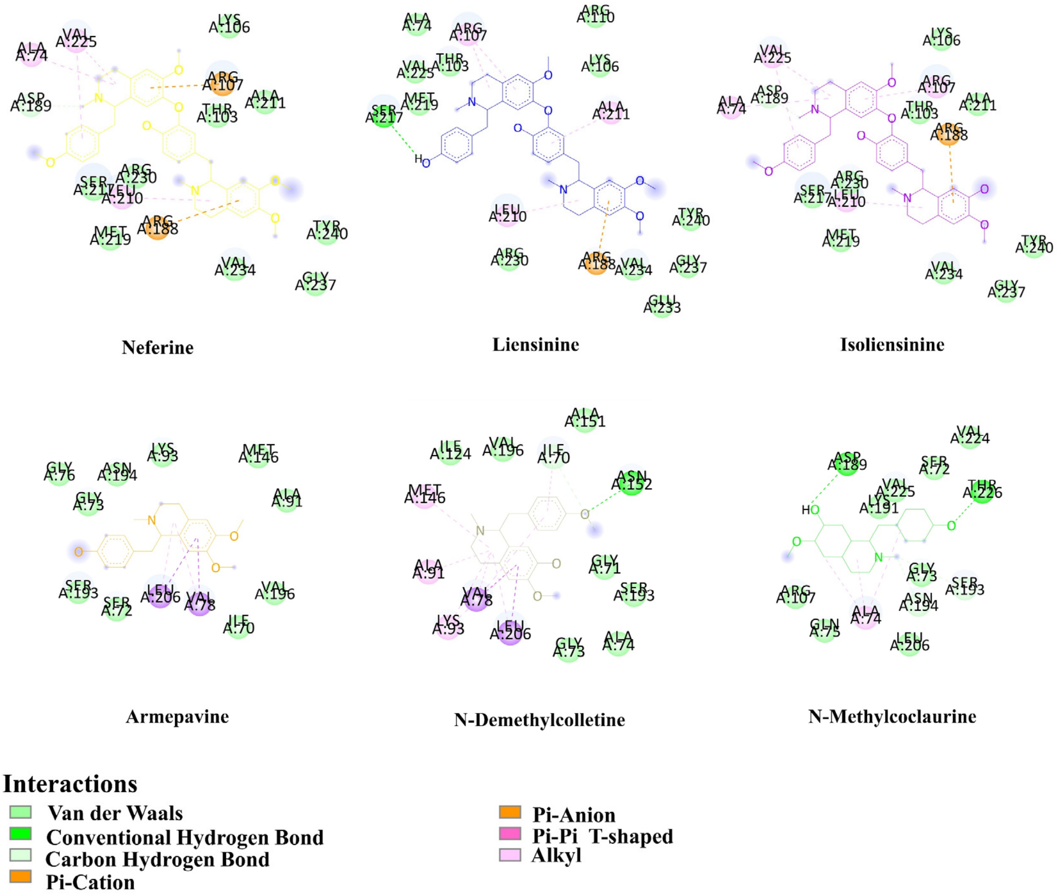


**Figure S2.** The interaction of Jun and six compounds.

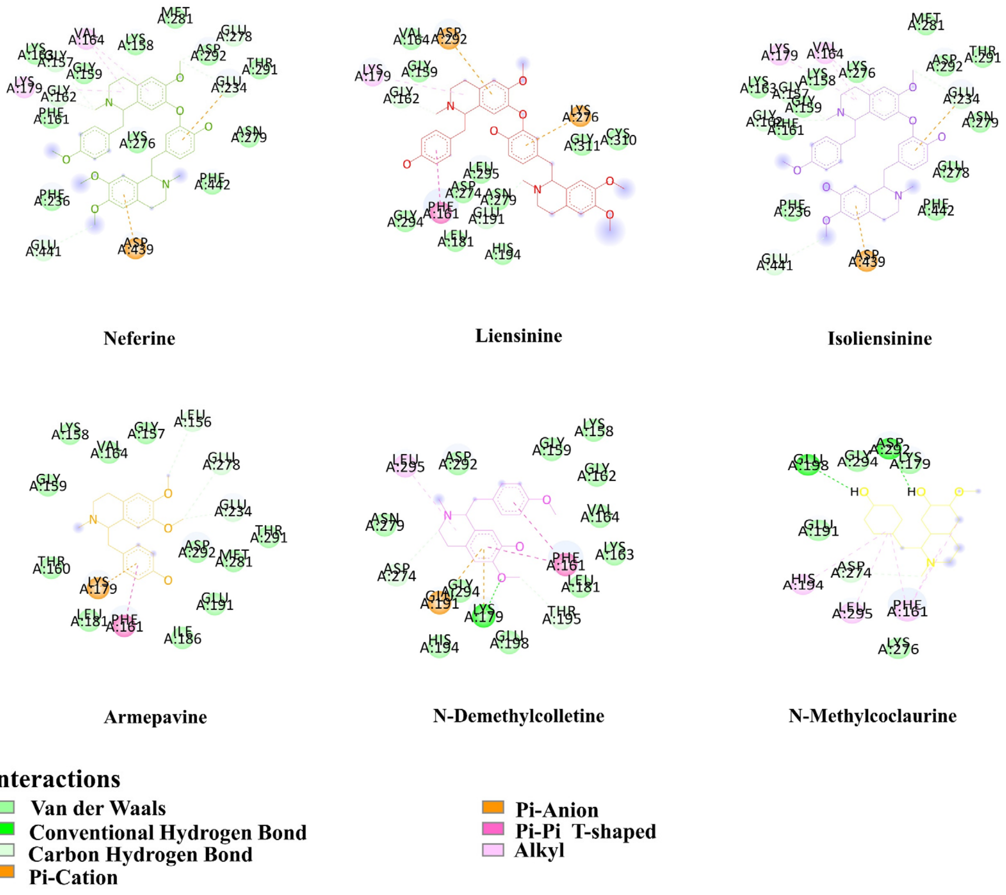


**Figure S3.** The interaction of AKT and six compounds.

**Table S1. Binding sites of six compounds for three targets.**

| Targets | Compounds | H-Bonds interacting residues | Van der waals interacting residues |
|---|---|---|---|
| mTOR | Neferine | | GLY1897,ASP1933,**GLN1937**,GLY2203, THR2207,**GLY2142**,THP2143,TYR2144, ASP2145,PRO2146,ASN2147,PHE2182, TYR2225 |
| | Liensinine | | LEU1936,ILE1939,TYP1974,**GLY2142**,THP2143,TYR2144,ARG2224,VAL2227,PRO2229 |
| | Isoliensinine | TRP2144,THP2207 | ASN1898,LEU1900,ILE1939,**GLN1937**,PRO1940,**GLY2142**,ASP2145,PRO2146,GLU2196,LEU2204,ARG2224,ALA2226,VAL2227,ILE2228 |
| | Armepavine | GLN1970 | ILE1939,ALA1971,ILE1973,TYR1974,PRO1975,ALA2139,**GLY2142**,THR2143,TYR2144,ARG2197,GLU2198,**GLN2200**,ARG2224,**MET2199**,ILE2228, |
| | N-Demethylcolletine | ARG2224 | **LEU1936**,GLU2196,**MET2199**,**GLN2200**, **GLY2203**,PRO2146 |
| | N-Methylcoclaurine | **GLY2142**, TYR2144 | **GLN1937**,PRO1940,GLN1970,TYR1974, ALA2139,THR2143,ASP2145,PRO2146 |
| | Control Ⅰ | ALA1971,VAL2227 | LEU1900,**LEU1936**,**GLN1937**,PRO1940, TYR1974,**GLY2142**,MET2199,**GLN2200**, **GLY2203**,ALA2226 |
| Jun | Neferine | | **THR103**,**LYS106**,ASP189,ALA211,SER217,MET219,**ARG230**,VAL234,GLY237,**TYR240** |
| | Liensinine | SER217 | ALA74,**THR103**,**LYS106**,ARG110,MET219,VAL225,**ARG230**,GLU233,VAL234, GLY237,**TYR240** |
| | Isoliensinine | | **THR103**,**LYS106**,ASP189,ALA211,SER217,MET219,**ARG230**,VAL234,GLY237,**TYR240** |
| | Armepavine | | ILE70,SER72,**GLY73**,GLY76,ALA91,LYS93,MET146,**SER193**,ASN194,VAL196 |
| | N-Demethylcolletine | ASN152 | ILE70,GLY71,**GLY73**,ALA74,ILE124,ALA151,**SER193**,VAL196 |
| | N-Methylcoclaurine | ASP189,THR226 | SER72,**GLY73**,GLN75,ARG107,LYS191, **SER193**,ASN194,LEU206,VAL224,VAL225 |
| | Control Ⅱ | ARG107 | ALA74,GLN75,ARG110,ASP189,LYS191,ASN194,GLY209,LEU210,ALA211,SER217,**ARG230** |

| | | | |
|---|---|---|---|
| AKT | Neferine | | GLY157,LYS158,**GLY159**,PHE161,GLY162,LYS163,GLU234,PHE236,LYS276,GLU278,ASN279,MET281,THR291,ASP292,GLU441,PHE442 |
| | Liensinine | | **GLY159**,GLY162,VAL164,LEU181,GLU191,HIS194,ASP274,ASN279,GLY294,LEU295,GLY311,CYS310 |
| | Isoliensinine | | GLY157,LYS158,**GLY159**,PHE161,GLY162,LYS163,GLU234,PHE236,LYS276,GLU278,ASN279,MET281,THR291,ASP292,GLU441,PHE442 |
| | Armepavine | | LEU156,GLY157,LYS158,**GLY159**,THR160,VAL164,LEU181,ILE186,GLU191,GLU234,GLU278,MET281,THR291,ASP292 |
| | N-Demethylcolletine | LYS179 | LYS158,**GLY159**,GLY162,LYS163,VAL164,LEU181,HIS194,THR195,GLU198,ASP274,ASN279,ASP292,GLY294 |
| | N-Methylcoclaurine | GLU198,ASP292 | GLY294,LYS179,LYS276,ASP274 |
| | Control Ⅲ | | GLY157,LYS158,**GLY159**,PHE161,GLY162,LYS163,LYS179,LEU181,HIS194,GLU234,GLU278,ASN279,THR291,ASP292,GLY294 |

Control Ⅰ: Adenosine-5'-diphosphate.

Control Ⅱ: 4-{[5-chloro-4-(1H-indol-3-yl)pyrimidin-2-yl]amino}-N-ethylpiperidine-1-carboxamide.

Control Ⅲ: (2S)-2-(4-chlorobenzyl)-3-oxo-3-[4-(7H-pyrrolo[2,3-d]pyrimidin-4-yl)piperazin-1-yl]propan-1-amine.
